# Characterization of impaired beta and alpha cell function in response to an oral glucose challenge in cystic fibrosis: a cross-sectional study

**DOI:** 10.3389/fendo.2023.1249876

**Published:** 2023-08-31

**Authors:** Bibi Uhre Nielsen, Inger Hee Mabuza Mathiesen, Rikke Møller, Rikke Krogh-Madsen, Terese Lea Katzenstein, Tacjana Pressler, James A. M. Shaw, Christian Ritz, Michael R. Rickels, Darko Stefanovski, Thomas Peter Almdal, Daniel Faurholt-Jepsen

**Affiliations:** ^1^ Cystic Fibrosis Centre Copenhagen, Department of Infectious Diseases, Copenhagen University Hospital - Rigshospitalet, Copenhagen, Denmark; ^2^ Centre for Physical Activity Research, Copenhagen University Hospital - Rigshospitalet, Copenhagen, Denmark; ^3^ Department of Infectious Diseases, Copenhagen University Hospital - Amager and Hvidovre, Copenhagen, Denmark; ^4^ Translational and Clinical Research Institute, Newcastle University, Newcastle upon Tyne, United Kingdom; ^5^ National Institute of Public Health, University of Southern Denmark, Copenhagen, Denmark; ^6^ Division of Endocrinology, Diabetes & Metabolism, Department of Medicine, and Institute for Diabetes, Obesity & Metabolism, University of Pennsylvania Perelman School of Medicine, Philadelphia, PA, United States; ^7^ Department of Clinical Studies - New Bolton Center, University of Pennsylvania School of Veterinary Medicine, Kennett Square, PA, United States; ^8^ Department of Endocrinology, Copenhagen University Hospital - Rigshospitalet, Copenhagen, Denmark

**Keywords:** cystic fibrosis, glucose tolerance, beta cells, alpha cells, hypoglycaemia

## Abstract

**Aims:**

The purpose of the study was to further elucidate the pathophysiology of cystic fibrosis (CF)-related diabetes (CFRD) and potential drivers of hypoglycaemia. Hence, we aimed to describe and compare beta cell function (insulin and proinsulin) and alpha cell function (glucagon) in relation to glucose tolerance in adults with CF and to study whether hypoglycaemia following oral glucose challenge may represent an early sign of islet cell impairment.

**Methods:**

Adults with CF (≥18 years) were included in a cross-sectional study using an extended (-10, -1, 10, 20, 30, 45, 60, 90, 120, 150, and 180 min) or a standard (-1, 30, 60, and 120 min) oral glucose tolerance test (OGTT). Participants were classified according to glucose tolerance status and hypoglycaemia was defined as 3-hour glucose <3.9 mmol/L in those with normal glucose tolerance (NGT) and early glucose intolerance (EGI).

**Results:**

Among 93 participants, 67 underwent an extended OGTT. In addition to worsening in insulin secretion, the progression to CFRD was associated with signs of beta cell stress, as the fasting proinsulin-to-insulin ratio incrementally increased (p-value for trend=0.013). The maximum proinsulin level (pmol/L) was positively associated with the nadir glucagon, as nadir glucagon increased 6.2% (95% confidence interval: 1.4-11.3%) for each unit increase in proinsulin. Those with hypoglycaemia had higher 60-min glucose, 120-min C-peptide, and 180-min glucagon levels (27.8% [11.3-46.7%], 42.9% [5.9-92.85%], and 80.3% [14.9-182.9%], respectively) and unaltered proinsulin-to-insulin ratio compared to those without hypoglycaemia.

**Conclusions:**

The maximum proinsulin concentration was positively associated with nadir glucagon during the OGTT, suggesting that beta cell stress is associated with abnormal alpha cell function in adults with CF. In addition, hypoglycaemia seemed to be explained by a temporal mismatch between glucose and insulin levels rather than by an impaired glucagon response.

## Introduction

The prevalence of cystic fibrosis (CF)-related diabetes (CFRD) is up to 40% among adults with CF (1). With increasing life-expectancy in CF (2), the complication of CFRD is expected to become even more common. Still, the pathophysiology leading to CFRD is not well understood, and thus more basic knowledge is needed to develop strategies to prevent and improve the treatment of CFRD.

The beta cell secretory capacity varies substantially in the adult CF population. Intriguingly, this seems to be irrespective of macroscopic pancreatic abnormalities (3), as the exocrine pancreatic tissue is replaced with fibrosis and fat in the vast majority of adults with CF (4, 5). Thus, somehow the endocrine tissue is differentially affected by this process and a substantial fraction of individuals with CF progress to insulin dependent CFRD with the mechanism being unclear.

Amongst potential mechanisms, it has been proposed that intra-islet inflammation (6), amyloid deposition and endoplasmic reticulum stress (7) may mediate progression to CFRD. These mechanisms of beta cell stress and dysfunction in CF may be evidenced by excessive secretion of the insulin precursor, proinsulin, as seen in type 2 diabetes (8). Thus, proinsulin-to-insulin ratio may be a suitable marker of beta cell stress. Consistently, previous studies have indicated that glucose intolerance in CF is associated with increased proinsulin levels (9–11). Furthermore, stress-induced pathways in the islets of Langerhans might also affect the alpha cells, which are responsible for glucagon secretion (6). A previous study reported reduced glucagon suppression in individuals with CF and glucose intolerance during an oral glucose tolerance test (OGTT) (12), suggesting that functions of other islet cell types are also affected by the CFRD pathology. However, this finding could simply be explained by insufficient insulin secretion, as insulin suppresses glucagon secretion physiologically (13). Hence, the association between beta and alpha cell dysfunction merits further investigation in CF.

Alterations in beta and alpha cell function might contribute to hypoglycaemia, which is frequently observed following ingestion of glucose or high glycaemic index foods in CF (14). Hypoglycaemia in CF may be caused by the mismatch between the rise in glucose and delayed insulin secretion from the beta cells (15). In addition, slow responding alpha cells with sub-optimal glucagon secretion might fail to compensate sufficiently to prevent post-prandial hypoglycaemia (16). Both theories suggest that post-prandial hypoglycaemia might be an early sign of islet dysfunction in CF.

To investigate potential indicators of beta and alpha cell dysfunction among adults with CF, we studied the hormonal secretion of beta cells (insulin and proinsulin) and alpha cells (glucagon) during an OGTT in individuals across the spectrum of glucose tolerance. Moreover, we compared beta and alpha cell function in individuals with CF who did and did not experience hypoglycaemia in order to examine whether the occurrence of hypoglycaemia represented an early sign of islet cell impairment.

## Methods

### Settings and participants

This was a cross-sectional study conducted at the Copenhagen CF Center. The inclusion criteria were age ≥18 years and a confirmed CF diagnosis. Except from individuals with lung-transplantation and pregnant women, all adult patients without CFRD and patients with CFRD, who had participated in prior research at our centre were invited to participate.

### Oral glucose tolerance test

Participants were fasted ≥8 hours before the OGTT and individuals with insulin therapy were requested to withhold short-acting insulin for 12 hours and long-acting insulin for 24 hours before the test. All participants drank 75 grams of glucose diluted in water. Depending on the personal preference, the participant underwent an extended 3-hour OGTT or a standard 2-hour OGTT. In the extended OGTT, a cannula for blood collection was inserted in an antecubital vein and blood was drawn 10 and 1 min prior to and 10, 20, 30, 45, 60, 90, 120, 150, and 180 min after glucose ingestion. In the standard OGTT, blood samples were collected 1 min prior and 30, 60, and 120 min after the glucose ingestion. Samples were stored on ice until centrifugation. Glucose, C-peptide, insulin (serum tubes) as well as intact proinsulin (EDTA tubes) were centrifuged at room temperature. Glucagon (BD™ P800^®^ tubes) was centrifuged at 4°C. After sample collection, centrifugation occurred within 1 hour and the samples were frozen (-80°C) immediately after. Glucose (702 module of Cobas 8000), C-peptide (801 module of Cobas 8000), insulin (801 module of Cobas 8000), proinsulin (TECOmedical Intact Proinsulin ELISA with Proinsulin Dynex DS2 ELISA analyser) and glucagon (Mercodia Glucagon ELISA with Glucagon Dynex DS2 ELISA analyser) were analysed with quantitative assays. All biochemistry analyses were performed all together at Exeter Clinical Laboratory (accredited by the United Kingdom Accreditation Service).

In line with previous protocols (11), participants were divided into groups with increasing severity of glucose intolerance; normal glucose tolerance (NGT): 1-hour glucose <8.6 mmol/L and 2-hour glucose <7.8 mmol/L, early glucose intolerance (EGI): 1-hour glucose ≥8.6 mmol/L and 2-hour glucose <7.8 mmol/L, impaired glucose tolerance (IGT): 2-hour glucose between 7.8-11.0 mmol/L and CFRD: 2-hour glucose ≥11.1 mmol/L. Homeostasis model assessment (HOMA-IR) and Matsuda index were used as markers of insulin resistance/sensitivity (17). HOMA-IR was calculated as (Insulin_fasting_ (μU/mL)*Glucose_fasting_ (mmol/L))/22.5 and Matsuda index as 10,000/(√[(Glucose_fasting_ (mg/dL)*Insulin_fasting_ (μU/mL))*(Glucose_mean_ (mg/dL)*Insulin_mean_ (μU/mL))]) using values from the standard OGTT. Among participants with NGT or EGI, hypoglycaemia and non-hypoglycaemia sub-groups were determined based on 3-hour glucose <3.9 mmol/L or ≥3.9 mmol/L, respectively. In addition, the hypoglycaemia threshold was reduced to 3.0 mmol/L in a sensitivity analysis (18). IGT and CFRD were excluded *a priori* in the hypoglycaemia analysis, given low incidence of post-OGTT low glucose in these groups. Demographic data and health status were obtained from the Danish CF Registry (age, sex, mutation class, faecal elastase, weight, height, lung function, modulator treatment and years on insulin treatment). Pancreas insufficiency was defined as faecal elastase ≤200 µg/g.

### Beta and alpha cell hormone secretion estimation using mathematical models

Insulin secretion rate and proinsulin appearance were estimated in WinSAAM version 3.3.0 (19). Insulin secretion rate was assessed using a modified minimal model by Breda et al. (20). In contrast to the insulin secretion model, which is unaffected by hepatic extraction, a proinsulin secretion model is not justified, as part of the proinsulin is extracted by the liver. Instead, we estimated the proinsulin appearance, which is a measure of the secreted proinsulin that passes the liver. Nevertheless, only a modest proportion of the proinsulin secretion is presumed to be extracted by the liver, as no difference in proinsulin levels in the hepatic and the systemic circulation could be detected in humans (21) and the hepatic extraction fraction for proinsulin was found to be constant and low (10-15 times lower than the hepatic extraction of insulin) (22). Hence, we assumed that most of the proinsulin secretion will pass the liver. Proinsulin was assumed to have a one compartment, first order elimination (23) with a half-life of 19 min (24), which is consistent with human studies (23). With these assumptions, proinsulin appearance could be estimated by deconvolution of proinsulin concentrations in plasma and a known one compartmental model. As proinsulin was only sampled at -1, 30, 60, 120, 180 min, we used interpolation with cubic splines to eliminate artificial spikes in the proinsulin appearance. The ratio of the area under the curve (AUC) of insulin secretion rate and proinsulin appearance was calculated for different phases of the OGTT (-10- (–1) min, 0-60 min, 60-120 min, and 120-180 min). The ratio was calculated for both AUCs above zero (total) and above baseline (incremental). As a surrogate measure for glucagon secretion, we used the glucagon concentrations with cubic spline interpolation, as glucagon has a short half-life (4.5 min) independent of glucose tolerance in non-CF individuals (25) and a stable hepatic extraction fraction around 20% (26). The glucagon secretion was divided into four phases ([fasting: -10, -1 min], [very early: 10 min], [intermediary: 20, 30, 60, 90, 120 min], and [late: 150, 180 min]). Only data from participants with an extended OGTT were included in the insulin secretion model, the proinsulin appearance model, the AUCs calculations and the glucagon model. To approximate maximum and minimum secretion in all participants, we generated estimates of the maximum concentrations (C_Max_) of glucose, C-peptide, and proinsulin as well as the nadir (C_Min_) of glucagon, respectively, with cubic spline interpolation between the four concentrations measures in the standard OGTT (-1, 30, 60, and 120 min).

### Statistical analyses

Baseline characteristics of the participants were summarized by medians and interquartile range (IQR) or proportions (%). A Chi-squared test was used to test whether females and males had different glucose tolerance distributions in our sample. Glucose concentrations, insulin secretion rate and proinsulin appearance during the OGTT were presented as mean and 95% confidence interval (95% CI) and the glucagon concentrations were plotted as median (IQR), as data were highly skewed. The relationship between proinsulin appearance and insulin secretion rate (time intervals between maximum levels (ΔT_Max_) and ratios of AUC), and time to nadir (T_Min_) of glucagon were compared in linear regression models across glucose tolerance groups. We assessed the correlation between glucose or insulin secretion rate and glucagon with Spearman’s correlation. Associations between glucose or insulin secretion rate and glucagon during four OGTT phases were assessed in linear mixed models with and without effect modification of glucose tolerance group and with random intercepts for each participant. Linear regression models were used to assess the associations between C_Max_ of glucose, C-peptide, and proinsulin vs. the logarithmic value of C_Min_ of glucagon. This model was further adjusted for age, sex, body mass index and exocrine pancreas function (insufficient/sufficient). The prevalence of hypoglycaemia in those with NGT and EGI was compared in a Chi-squared test. Glucose and different pancreas hormone concentrations (logarithmically transformed) in individuals with hypoglycaemia were compared to individuals without hypoglycaemia in linear mixed models. The linear mixed model accounted for individual variation with random intercepts and was also adjusted for age, sex, body mass index and exocrine pancreas function (insufficient/sufficient). All models, including the mixed models, used robust standard errors (27). Robust standard errors reference to the estimation procedure, which provides robust estimates of the variance including standard errors. This was used to correct for small departures of the outcome from normality. Beta coefficients from models with logarithmic transformed outcomes were back transformed into percentage changes. The estimates of C_Max_ of glucose, C-peptide, and proinsulin and C_Min_ of glucagon were validated against the modelled estimates from the extended OGTTs with Spearman’s correlation. P-values <0.05 (two-tailed) were considered significant and R x64 (version 4.2.2) and RStudio (version 1.2.5001) were used for the statistical analyses.

### Ethical considerations

Ethical approval (H-19085530) was obtained from the Regional Committee on Health Research Ethics prior to enrolment of participants and all participants signed the consent form before entering the study.

## Results

### Baseline characteristics

Between August 2020 and January 2021, 93 out of 159 eligible adults with CF participated in the study. Sixty-seven participants (72%) underwent the extended OGTT. Most participants were pancreas insufficient and had severe mutations. Ninety-one (98%) had at least one F508del mutation and 60 (65%) were homozygous. Out of the 93 participants, 21 (23%) presented with NGT, 30 (32%) with EGI, 14 (15%) with IGT and 28 (30%) with CFRD (**Table 1**). The prevalence of the different glucose tolerance groups was comparable for females and males in our sample (p-value=0.499).

**Table 1:** Characteristics of 93 adults with cystic fibrosis.

	n = 93
Demographics	
Age (years)	31 (24.0, 40.0)
Female	33 (35%)
Lung function	
FEV_1_%	79.1 (56.1, 99.0)
Mutations^a^	
Severe mutation class (I-III)	82 (88%)
Mild mutation class (IV-VI)	11 (12%)
Nutritional status	
Pancreas insufficient	85 (91%)
Body mass index (kg/m²)	22.6 (21.0, 24.3)
Glucose tolerance	
Normal glucose tolerance	21 (23%)
Early glucose intolerance	30 (32%)
Impaired glucose tolerance	14 (15%)
Cystic fibrosis-related diabetes	28 (30%)
Insulin treatment^b^	
Treated with insulin	13 (14%)
Insulin treatment period (years)^c^	14.0 (9.0, 23.0)
Modulator treatment	
None	27 (29%)
Lumacaftor/ivacaftor	2 (2%)
Tezacaftor/ivacaftor	51 (55%)
Elexacaftor/tezacaftor/ivacaftor	13 (14%)

Data is shown as median (interquartile range) or n (%).

a 91 (98%) had at least one F508del mutation and 60 (65%) were homozygous.

b Only insulin was prescribed as diabetic medication in the study cohort.

c Years of insulin treatment among participants, who were treated with insulin. FEV_1_%, percent predicted forced expiratory volume in one second.

### Hormone secretion by glucose tolerance groups

**Figure 1:**
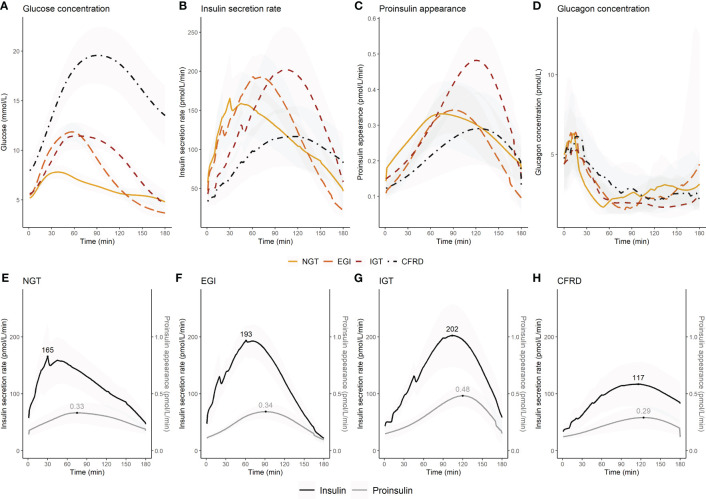
Changes in glucose concentration, insulin secretion rate, proinsulin appearance, and glucagon concentration during an extended oral glucose tolerance test in 67 adults with cystic fibrosis by glucose tolerance status. The upper panel shows **(A)** glucose concentration, **(B)** insulin secretion rate, **(C)** proinsulin appearance, and **(D)** glucagon concentration for each glucose tolerance group. The lower panel shows insulin secretion rate and proinsulin appearance by glucose tolerance group **(E–H)**. In the lower panel the maximum insulin secretion rate and proinsulin appearance are indicated. Insulin secretion rate **(B, E–H)** and proinsulin appearance **(C, E–H)** were presented with means (95% confidence interval) for each minute. Glucagon concentrations were presented as median (interquartile range) for each minute **(D)**. Glucagon was not assessed in five individuals due to delivery failure of the samples. NGT, normal glucose tolerance; EGI, early glucose intolerance; IGT, impaired glucose tolerance; CFRD, cystic fibrosis-related diabetes.

Glucose levels, insulin secretion rate, proinsulin appearance and glucagon concentrations stratified by glucose tolerance during the OGTT are presented in dynamic plots (**Figures 1A–D**). **Figure 1** shows that glucose intolerance was characterised by a delayed insulin secretion, but only those with CFRD had a diminished overall insulin secretion (**Figure 1B**). Time to peak for the proinsulin appearance was less affected by glucose tolerance, however proinsulin secretion was highest in those with IGT (**Figure 1C**). The figure also shows comparable peak glucagon concentration over the first 30 minutes post-glucose ingestion (**Figure 1D**), but increasing time to glucagon nadir with worsening glucose tolerance (C_Min_) (**Supplementary Figure 1**).

**Figure 2:**
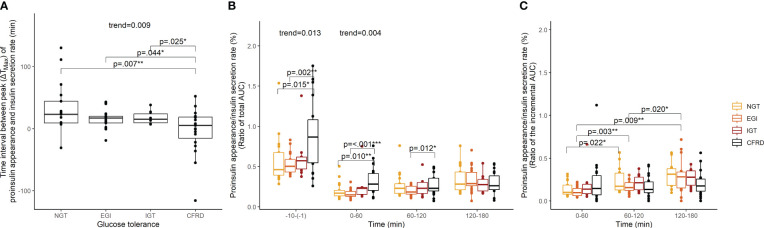
Proinsulin appearance relative to insulin secretion rate assessed with time interval between maximum values (ΔT_Max_) and ratios of area under the curve among 67 adults with cystic fibrosis during an extended oral glucose tolerance test. Proinsulin appearance relative to insulin secretion rate by glucose tolerance group assessed with **(A)** time interval between the maximum values, **(B)** the ratio of the total area under the curves, and **(C)** the ratio of the incremental area under the curves. P-values and p-values for trend were calculated in linear models using robust standard error. NGT, normal glucose tolerance; EGI, early glucose intolerance; IGT, impaired glucose tolerance; CFRD, cystic fibrosis-related diabetes; ΔT_Max_, time interval between maximum values; AUC, area under the curve. *p<0.05, **p<0.01, ***p<0.001.

The time of peak and magnitude of insulin secretion rate and proinsulin appearance were plotted for each glucose tolerance group (**Figures 1E–H**). We assessed the difference in the time interval between maximum insulin secretion rate and proinsulin appearance to indicate whether glucose stimulated proinsulin-to-insulin secretion ratio differed between groups. We found that the time interval between maximum insulin secretion rate and proinsulin appearance was shorter with increasing impairment in glucose tolerance (p-value for trend=0.009) (**Figure 2A**). We also found that the fasting proinsulin appearance/insulin secretion rate ratio (PA/ISR) increased across the glucose tolerance groups from NGT to CFRD (p-value for trend=0.013) (**Figure 2B**). However, during the OGTT the incremental PA/ISR did not vary across glucose tolerance groups (**Figure 2C**). Nevertheless, the incremental PA/ISR was lower during 0-60 min compared to 60-120 min among those with NGT and EGI, but not among those with IGT and CFRD.

### Time dependent correlations between insulin secretion and glucagon

**Figure 3:**
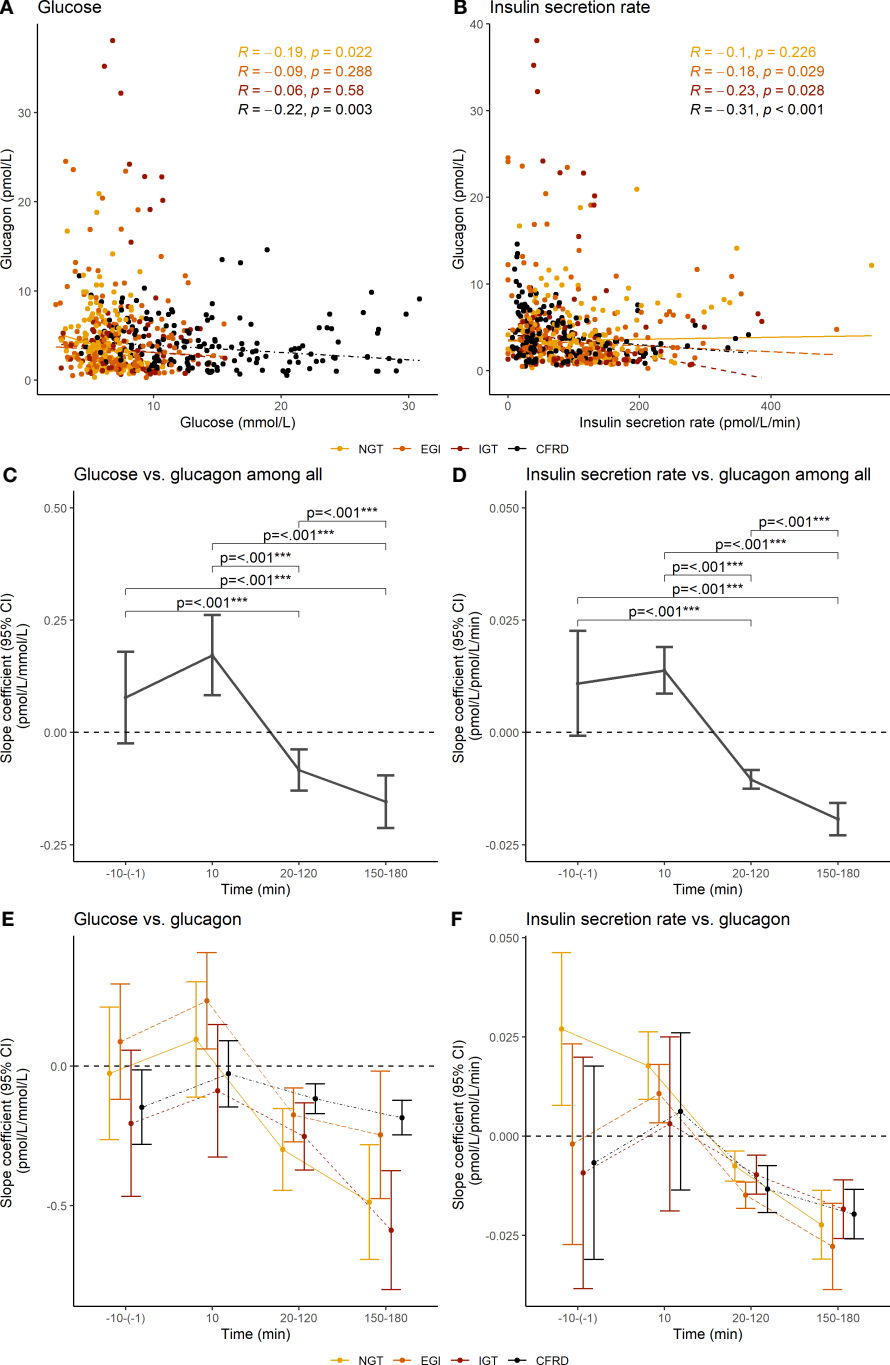
Overall correlations and segmental slope coefficients between glucose concentration and insulin secretion rate vs. glucagon concentration during an extended oral glucose tolerance test among 62 adults with cystic fibrosis. The upper panel shows Spearman’s correlations coefficients between **(A)** glucose concentration and **(B)** the insulin secretion rate and glucagon concentration. The middle and lower panel shows the slope coefficients and 95% confidence interval between glucose concentration **(C, E)** and insulin secretion rate **(D, F)** and glucagon concentration at different phases during the oral glucose tolerance test, with the lower panel **(E, F)** showing the results stratified by glucose tolerance. Slope coefficients were calculated with mixed models using robust standard error. Glucagon was not assessed in five individuals due to delivery failure of the samples. NGT, normal glucose tolerance; EGI, early glucose intolerance; IGT, impaired glucose tolerance; CFRD, cystic fibrosis-related diabetes; 95% CI, 95% confidence interval. ***p<0.001.

NGT and CFRD, but not EGI and IGT, presented with an overall negative correlation between glucose and glucagon. EGI, IGT, and CFRD, but not NGT, had an overall negative correlation between insulin secretion rate and glucagon (**Figures 3A, B**). Further investigation showed that the association between glucose and glucagon and between insulin secretion rate and glucagon were positive at 10 minutes, but negative between 20-180 min (**Figures 3C, D**). The associations did not differ between glucose tolerance groups (**Figures 3E, F**).

### Associations between beta and alpha cell hormone secretion

Among all participants, the maximum level (C_Max_) of glucose and C-peptide were not associated with the minimum level (C_Min_) of glucagon. However, the maximum level of proinsulin (C_Max_) was positively associated with the minimum level (C_Min_) of glucagon (**Table 2**). The coefficient of determination (R^2^) between modelled estimates from the extended OGTTs and C_Max_ and C_Min_ from the standard OGTTs were between 0.72-0.99 (**Supplementary Figure 2**).

**Table 2:** Associations between beta cell function and nadir concentration of glucagon in 87 adults with cystic fibrosis during a standard oral glucose tolerance test.

	Unadjusted				Adjusted			
	%	95% CI		p-value	%	95% CI		p-value
Age (years)	-0.4	-2.2	1.5	0.702	-0.4	-2.3	1.5	0.644
Female	-36.1	-56.1	-7.0	**0.020**	-36.4	-55.5	-9.2	**0.014**
Body mass index (kg/m²)	5.2	0.7	9.8	**0.022**	7.0	-0.2	14.7	0.055
HOMA-IR	8.0	-3.4	20.6	0.174	3.2	-8.5	16.2	0.607
Matsuda index	-0.9	-4.4	2.8	0.637	0.1	-3.2	3.6	0.937
Pancreas insufficient	-19.0	-52.9	39.5	0.443	34.7	-39.6	200.6	0.462
Glucose C_Max_ (mmol/L)	0.7	-2.8	4.3	0.701	1.4	-1.9	4.9	0.406
C-peptide C_Max_ (pmol/L)	-0.001	-0.02	0.02	0.896	-0.01	-0.03	0.01	0.401
Proinsulin C_Max_ (pmol/L)	5.8	1.1	10.7	**0.016**	6.2	1.4	11.3	**0.011**

Data is based on back transformed beta coefficients (% and 95% confidence intervals) estimated with linear regression models with robust standard error. Adjusted models were adjusted for age (years), sex (male or female), body mass index (kg/m²) and exocrine pancreas function (faecal elastase ≤ or >200 µg/g). C_Max_ of glucose, C-peptide, and proinsulin and C_Min_ (nadir) of glucagon were calculated from cubic spline interpolation between 4 time points (-1, 30, 60, and 120 min). Glucagon was not assessed in six individuals due to delivery failure of the samples. HOMA-IR Homeostasis model assessment; C_Max_, maximum concentration; C_Min_, minimum/nadir concentration; 95% CI, 95% confidence interval.

Bold values indicate statistical significance at the p<0.05 level.

### Levels of beta and alpha cell hormones in individuals with hypoglycaemia

Among those with either NGT or EGI, 37 (73%) had a 3-hour blood sample. Three (17.6%) out of 17 with NGT had hypoglycaemia, while 12 (60%) out of 20 with EGI had hypoglycaemia. The prevalence of hypoglycaemia differed significantly between NGT and EGI (p=0.023). At 60 minutes, participants with hypoglycaemia had 27.8% higher glucose levels (95% CI [11.3; 46.7%], p<0.001), but similar C-peptide levels, compared to non-hypoglycaemia. At 120 minutes, glucose levels were comparable, but participants with hypoglycaemia had higher C-peptide (42.9%, 95% CI [5.9; 92.8%], p=0.019) and proinsulin levels (32.5%, 95% CI [3.8; 69.2%], p=0.024). Throughout the OGTT, hypoglycaemia was not associated with the total PA/ISR. Glucagon levels were elevated after 180 min in those with hypoglycaemia compared to those without hypoglycaemia (80.3%, 95% CI [14.9; 182.9%], p=0.010) **(** **Table 3** **)**. When the threshold for hypoglycaemia was reduced to 3.0 mmol/L, only four were categorized as having hypoglycaemia and they were all in the group with EGI. The estimates were similar with the reduced threshold (**Supplementary Table 1**).

**Table 3:** Pancreas hormones in participants with hypoglycaemia reported as percentage difference relative to participants with non-hypoglycaemia in 37 adults with cystic fibrosis and either normal glucose tolerance or early glucose intolerance during an extended oral glucose tolerance test.

	n	Adjusted			
		%	95% CI		p-value
Glucose (mmol/L)					
60 min	37	27.8	11.3	46.7	**<0.001**
120 min	37	-7.9	-19.8	5.8	0.244
180 min	37	-33.2	-41.8	-23.3	**<0.001**
Insulin (pmol/L)					
60 min	37	24.2	-22.2	98.2	0.364
120 min	37	48.3	-6.1	134.3	0.091
180 min	37	-27.3	-54.3	15.5	0.177
C-peptide (pmol/L)					
60 min	37	20.6	-11.0	63.6	0.227
120 min	37	42.9	5.9	92.8	**0.019**
180 min	37	-7.1	-31.2	25.5	0.630
Proinsulin (pmol/L)					
60 min	37	9.2	-14.5	39.4	0.482
120 min	37	32.5	3.8	69.2	**0.024**
180 min	37	-1.3	-22.7	26.0	0.913
Total proinsulin/insulin ratio (%)					
0-60 min	37	-8.2	-28.2	17.3	0.494
60-120 min	37	-5.6	-26.2	20.6	0.643
120-180 min	37	21.5	-5.0	55.3	0.120
Glucagon (pmol/L)					
60 min	32	-2.7	-37.7	52.0	0.905
120 min	32	-11.1	-43.1	38.9	0.607
180 min	32	80.3	14.9	182.9	**0.010**

Percentage difference in pancreas hormones in participants with hypoglycaemia (glucose_180 min_<3.9 mmol/L) relative to participants with non-hypoglycaemia. Data is based on back transformed beta coefficients (% and 95% confidence intervals) estimated with linear mixed models with time as factor and logarithmic transformed glucose concentrations/pancreas hormones. The model included robust standard errors and were adjusted for age (years), sex (male or female), body mass index (kg/m²) and exocrine pancreas function (faecal elastase ≤ or >200 µg/g). Total proinsulin/insulin ratio was calculated using the area under the curve of proinsulin appearance and insulin secretion rate. Glucagon was not assessed in five individuals due to delivery failure of the samples. Bold values indicate statistical significance at the p<0.05 level.

## Discussion

### Main findings

To elucidate islet pathophysiology underlying the development of CFRD, we studied beta and alpha cell function across the spectrum of glucose tolerance in CF. Our results demonstrate that those with EGI already manifest impaired early-phase insulin secretion, confirming prior investigation (11). We further found increased PA/ISR at fasting in those with CFRD, but during the glucose challenge the ratio was less impacted. Moreover, glucagon secretion was negatively associated with insulin in the late phase of the OGTT, which might explain the delayed suppression of glucagon in those with glucose intolerance. However, neither glucose nor insulin levels predicted the glucagon nadir, while the peak concentration of proinsulin was positively associated with the nadir level of glucagon, suggesting a possible relationship between beta and alpha cell dysfunction in the CF islet. Hypoglycaemia was more common in those with EGI compared to NGT where a delay in early-phase insulin secretion was associated with an increased insulin secretion in the late phase of the OGTT as suggested by a prior report (28).

### Glucose intolerance was linked to increased fasting proinsulin-to-insulin ratio, but less related to the glucose stimulated ratio

The proinsulin-to-insulin ratio seems to be a good general indicator of beta cell stress, as previous studies reported increased proinsulin-to-insulin ratios in CF and non-CF subjects with insufficient insulin secretion (9–11, 29, 30). Consistently, we found a reduction in the time interval between the peaks of the insulin secretion rate and proinsulin appearance with severity in glucose intolerance, which suggests that the glucose stimulated proinsulin-to-insulin secretion ratio changes with the progression towards CFRD. In addition, we found an elevated fasting PA/ISR in CFRD, which might indicate changes in constitutive insulin secretion from immature granules (31). However, our results indicate that the difference in PA/ISR between groups was less evident during the OGTT, as the secretion ratio above baseline showed no significant variation between the glucose tolerance groups. Still, these findings support that in addition to absolute beta cell loss, dysfunctional beta cells also play a role in the progression of abnormal glucose tolerance towards diabetes in CF. It is possible that the dysfunctional phenotype is an end-stage consequence of other pancreas pathology, i.e., inflammation or peri-islet fibrosis affecting blood flow and resulting in a delayed response of the beta cells in sensing fluctuations in glucose concentrations. Nevertheless, interventions that could relieve some of the beta cell stress may be of clinical relevance in CFRD.

### The correlation between insulin and glucagon secretion is time dependent

As non-CF studies have shown that insulin secretion suppresses glucagon secretion through intra-islet signalling (13), we did not expect to find an increased glucagon secretion and positive associations with both glucose levels and insulin secretion after 10 min of the OGTT. Though an early peak of glucagon has been seen following oral glucose challenge in non-CF studies (32–34), we are not aware of other studies evaluating this in CF. Nevertheless, a very early positive association between insulin and glucagon has previously been observed in non-CF individuals with abnormal glucose tolerance (35). A possible explanation is that early aberrant glucagon secretion is explained completely or partly by synthesis and secretion of glucagon by intestinal endocrine cells (36). Indeed, pancreatectomized patients exhibit paradoxically increased glucagon secretion in response to oral glucose (37) and glucagon has insulinotropic effects, similar to the incretin effect of glucagon-like-peptide-1 (38). However, whether enteric-derived glucagon might also support islet beta cell function in CF is still unknown.

### Increased proinsulin secretion was associated with increased glucagon secretion

*A priori*, we hypothesized that CFRD would have higher glucagon secretion during the OGTT compared to NGT, in line with the insufficient intra-islet suppression theory and findings from non-CF studies (39) and CF studies (12). Intriguingly, the nadir glucagon levels were not higher in those with low insulin secretion or high glucose levels, but the lowest glucagon levels were higher in those with high proinsulin levels. The elevation in nadir glucagon with increasing proinsulin secretion supports an intra-islet pancreatic defect affecting both beta and alpha cell functional phenotype. A *post mortem* tissue study in CF pancreases, has shown that peri-islet fibrosis was associated with having an increased number of alpha cells with an altered phenotype (anomalously expressing the mesenchymal marker, vimentin) (40). This suggests that changes in the islet environment may lead to endocrine cell dysfunction affecting both beta and alpha cells. As this explanation is speculative, more research is needed to prove any direct correlation between intrinsic beta and alpha cell impairment in CF.

### Delayed insulin secretion and a compensatory glucagon secretion were related to hypoglycaemia

The gradual progression to CFRD makes it possible to study early islet impairment and related glucose intolerance in stages before CFRD. The current study demonstrated that reactive hypoglycaemia following an oral glucose challenge was linked to early impairment of glucose tolerance. Our study agrees with previous work (15, 28) showing that high levels of glucose at 60 min related to impaired early-phase insulin secretion and consequently high levels of insulin at 120 min were seen in those with hypoglycaemia. Hence, a shift in the dynamics of insulin secretion seems to best explain the occurrence of late post-prandial or oral glucose load hypoglycaemia in CF. Furthermore, we found compensatory increased glucagon secretion in those with hypoglycaemia, in contrast to what has been reported previously (16, 28). Both Aithken et al. (16) and Kilberg et al. (28) showed no change in the 180 min glucagon response after an OGTT when comparing those with and without hypoglycaemia. However, it should be noted that these studies also included participants with IGT and higher insulin secretion from 120-180 min (28), which might have suppressed the glucagon response despite the concurrent hypoglycaemia. In our study, those with EGI had a low insulin secretion in the late phase of the OGTT, which may have allowed a glucagon response. Although we cannot reject that the hypoglycaemic glucagon response was delayed and/or suboptimal in CF, lack of glucagon response does not seem to explain the reactive hypoglycaemia seen in those with NGT and EGI. In summary, this study holds little evidence that an abnormal glucagon response has a clinical impact in CFRD, although the function of the alpha cells seems to be affected by the pathophysiology of CFRD.

### Strengths and limitations

A strength of this study is the inclusion of a relatively large sample size of well-characterised individuals with CF who were clinically stable at the time of metabolic testing. Nevertheless, the absolute numbers in each group were small, which increases the risk for type 1 and type 2 errors. The models developed to estimate proinsulin appearance and glucagon secretion independent of other hormones are novel, with no other published models available for comparison. Therefore, we used simple assumptions to derive markers of proinsulin and glucagon secretion, but some errors related to the hepatic extraction and variation in clearance cannot be disregarded completely. This study was also limited by the use of two different study protocols (i.e., standard and extended) according to participant preference. However, all models took this into account by either excluding the standard OGTTs or excluding samples not in the standard OGTT. Finally, this cross-sectional study was not designed to identify causes of CFRD and hypoglycaemia.

## Conclusion

In conclusion, the progression from NGT to CFRD is accompanied by worsening insulin secretion and associated with signs of beta cell stress and dysfunction including increased fasting PA/ISR. Although the alpha cell responsiveness to glucose and insulin secretion fluctuated over the course of the OGTT, the responsiveness was equal in all glucose tolerance groups. At the same time, the maximum proinsulin level was positively associated with the nadir glucagon level, which suggests a connection between beta cell and alpha cell dysfunction in CF. Furthermore, delayed beta-cell insulin secretion rather than alpha cell dysfunction likely explains the occurrence of post-oral glucose challenge hypoglycaemia seen early in the development of glucose intolerance in CF.

## Data availability statement

The datasets presented in this article are not readily available because they contain sensitive personal data. Requests to access the datasets should be directed to the corresponding author.

## Ethics statement

The studies involving humans were approved by Regional Committee on Health Research Ethics in the Capital Region of Denmark. The studies were conducted in accordance with the local legislation and institutional requirements. The participants provided their written informed consent to participate in this study.

## Author contributions

Conceived and designed the analyses: BN, JS, DF-J, MR, TA, IM, TK, TP, RK-M, and RM. Collected the data: BN and RM. Performed the analysis: BN, CR, and DS. Wrote the paper: BN, DF-J, TA, JS, IM, CR, TK, TP, RK-M, RM, MR, and DS. All authors contributed to the article and approved the submitted version.

## References

[1] Moran A Dunitz J Nathan B Saeed A Holme B Thomas W. Cystic fibrosis-related diabetes: Current trends in prevalence, incidence, and mortality. Diabetes Care (2009) 32:1626–31. doi: 10.2337/dc09-0586 PMC2732133 19542209

[2] Burgel PR Bellis G Olesen HV Viviani L Zolin A Blasi F. Future trends in cystic fibrosis demography in 34 European countries. Eur Respir J (2015) 46:133–41. doi: 10.1183/09031936.00196314 25792639

[3] Norris AW Ode KL Merjaneh L Sanda S Yi Y Sun X. Survival in a bad neighborhood: Pancreatic islets in cystic fibrosis. J Endocrinol (2019) 241:R35–50. doi: 10.1530/JOE-18-0468 PMC6675675 30759072

[4] Löhr M Goertchen P Nizze H Gould NS Gould VE Oberholzer M. Cystic fibrosis associated islet changes may provide a basis for diabetes - An immunocytochemical and morphometrical study. Virchows Arch A Pathol Anat Histopathol (1989) 414:179–85. doi: 10.1007/BF00718598 2492695

[5] Bogdani M Blackman SM Ridaura C Bellocq JP Powers AC Aguilar-Bryan L. Structural abnorMalities in islets from very young children with cystic fibrosis may contribute to cystic fibrosis-related diabetes. Sci Rep (2017) 7:17231. doi: 10.1038/s41598-017-17404-z 29222447 PMC5722914

[6] Hull RL Gibson RL McNamara S Deutsch GH Fligner CL Frevert CW. Islet interleukin-1β immunoreactivity is an early feature of cystic fibrosis that may contribute to β-cell failure. Diabetes Care (2018) 41:823–30. doi: 10.2337/dc17-1387 PMC5860832 29437698

[7] Kelly A Sheikh S. Cystic fibrosis-related diabetes: links, challenges, and future directions. Res Rep Endocr Disord (2015) 5:157. doi: 10.2147/rred.s68278

[8] Kahn SE Halban PA. Release of incompletely processed proinsulin is the cause of the disproportionate proinsulinemia of NIDDM. Diabetes (1997) 46:1725–32. doi: 10.2337/DIAB.46.11.1725 9356018

[9] Hartling SG Garne S Binder C Heilmann C Petersen W Petersen KE. Proinsulin, insulin, and C-peptide in cystic fibrosis after an oral glucose tolerance test. Diabetes Res (1988) 7:165–9. 3042255

[10] Hamdi I Green M Shneerson JM Palmer CR Hales CN. Proinsulin, proinsulin intermediate and insulin in cystic fibrosis. Clin Endocrinol (Oxf) (1993) 39:21–6. doi: 10.1111/j.1365-2265.1993.tb01746.x 8348704

[11] Nyirjesy SC Sheikh S Hadjiliadis D De Leon DD Peleckis AJ Eiel JN. β-Cell secretory defects are present in pancreatic insufficient cystic fibrosis with 1-hour oral glucose tolerance test glucose ≥155 mg/dL. Pediatr Diabetes (2018) 19:1173–82. doi: 10.1111/pedi.12700 PMC6364976 29885044

[12] Lanng S Thorsteinsson B Roder ME Orskov C Holst JJ Nerup J. Pancreas and gut hormone responses to oral glucose and intravenous glucagon in cystic fibrosis patients with normal, impaired, and diabetic glucose tolerance. Acta Endocrinol (Copenh) (1993) 128:207–14. doi: 10.1530/acta.0.1280207 8480468

[13] Meier JJ Kjems LL Veldhuis JD Lefèbvre P Butler PC. Postprandial suppression of glucagon secretion depends on intact pulsatile insulin secretion: Further evidence for the intraislet insulin hypothesis. Diabetes (2006) 55:1051–6. doi: 10.2337/diabetes.55.04.06.db05-1449 16567528

[14] Hirsch IB Janci MM Goss CH Aitken ML. Hypoglycemia in adults with cystic fibrosis during oral glucose tolerance testing. Diabetes Care (2013) 36:121–2. doi: 10.2337/dc12-1859 PMC3714472 23881975

[15] Kilberg MJ Sheikh S Stefanovski D Kubrak C De Leon DD Hadjiliadis D. Dysregulated insulin in pancreatic insufficient cystic fibrosis with post-prandial hypoglycemia. J Cyst Fibros (2020) 19:310–5. doi: 10.1016/j.jcf.2019.07.006 PMC7007375 31402215

[16] Aitken ML Szkudlinska MA Boyko EJ Ng D Utzschneider KM Kahn SE. Impaired counterregulatory responses to hypoglycaemia following oral glucose in adults with cystic fibrosis. Diabetologia (2020) 63:1055–65. doi: 10.1007/s00125-020-05096-6 PMC7150633 31993716

[17] Muniyappa R Lee S Chen H Quon MJ. Current approaches for assessing insulin sensitivity and resistance in *vivo*: advantages, limitations, and appropriate usage. Am J Physiol - Endocrinol Metab (2008) 294:15–26. doi: 10.1152/ajpendo.00645.2007 17957034

[18] Heller SR. Glucose concentrations of less than 3.0 mmol/L (54 mg/dL) should be reported in clinical trials: a joint position statement of the American diabetes association and the European association for the study of diabetes. Diabetes Care (2017) 40:155–7. doi: 10.2337/dc16-2215 27872155

[19] Stefanovski D Moate PJ Boston RC. WinSAAM: a windows-based compartmental modeling system. Metabolism (2003) 52:1153–66. doi: 10.1016/s0026-0495(03)00144-6 14506622

[20] Breda E Cavaghan MK Toffolo G Polonsky KS Cobelli C. Oral glucose tolerance test minimal model indexes of β-cell function and insulin sensitivity. Diabetes (2001) 50:150–8. doi: 10.2337/diabetes.50.1.150 11147781

[21] Henriksen JH Tronier B Bülow JB. Kinetics of circulating endogenous insulin, C-peptide, and proinsulin in fasting nondiabetic man. Metabolism (1987) 36:463–8. doi: 10.1016/0026-0495(87)90044-8 3553849

[22] Rubenstein AH Pottenger LA Mako M Getz GS Steiner DF. The metabolism of proinsulin and insulin by the liver. J Clin Invest (1972) 51:912–21. doi: 10.1172/JCI106886 PMC302205 5014618

[23] Tura A Pacini G Kautzky-Willer A Ludvik B Prager R Thomaseth K. Basal and dynamic proinsulin-insulin relationship to assess β-cell function during OGTT in metabolic disorders. Am J Physiol - Endocrinol Metab (2003) 285:155–62. doi: 10.1152/ajpendo.00104.2002 12670835

[24] Stoll RW Touber JL Winterscheid LC Ensinck JW Williams RH. Hypoglycemic activity and immunological half-life of porcine insulin and proinsulin in baboons and swine. Endocrinology (1971) 88:714–7. doi: 10.1210/endo-88-3-714 5541306

[25] Grøndahl MFG Lund AB Bagger JI Petersen TS Wewer Albrechtsen NJ Holst JJ. Glucagon clearance is preserved in type 2 diabetes. Diabetes (2022) 71:73–82. doi: 10.2337/db21-0024 34702780

[26] Ishida T Chap Z Chou J Lewis R Hartley C Entman M. Differential effects of oral, peripheral intravenous, and intraportal glucose on hepatic glucose uptake and insulin and glucagon extraction in conscious dogs. J Clin Invest (1983) 72:590–601. doi: 10.1172/JCI111007 6348094 PMC1129217

[27] Mansournia MA Nazemipour M Naimi AI Collins GS Campbell MJ. Reflection on modern methods: Demystifying robust standard errors for epidemiologists. Int J Epidemiol (2021) 50:346–51. doi: 10.1093/ije/dyaa260 33351919

[28] Kilberg MJ Harris C Sheikh S Stefanovski D Cuchel M Kubrak C. Hypoglycemia and islet dysfunction following oral glucose tolerance testing in pancreatic-insufficient cystic fibrosis. J Clin Endocrinol Metab (2020) 105:3179–89. doi: 10.1210/clinem/dgaa448 PMC7755140 32668452

[29] Breuer TGK Menge BA Banasch M Uhl W Tannapfel A Schmidt WE. Proinsulin levels in patients with pancreatic diabetes are associated with functional changes in insulin secretion rather than pancreatic β-cell area. Eur J Endocrinol (2010) 163:551–8. doi: 10.1530/EJE-10-0330 20679359

[30] Leahy JL Halban PA Weir GC. Relative hypersecretion of proinsulin in rat model of NIDDM. Diabetes (1991) 40:985–9. doi: 10.2337/diab.40.8.985 1860563

[31] Hou JC Min L Pessin JE. Insulin granule biogenesis, trafficking and exocytosis. Vitam Horm (2009) 80:473–506. doi: 10.1016/S0083-6729(08)00616-X 19251047 PMC4324607

[32] Pepino MY Tiemann CD Patterson BW Wice BM Klein S. Sucralose affects glycemic and hormonal responses to an oral glucose load. Diabetes Care (2013) 36:2530–5. doi: 10.2337/dc12-2221 PMC3747933 23633524

[33] Mumme L Breuer TGK Rohrer S Schenker N Menge BA Holst JJ. Defects in a-cell function in patients with diabetes due to chronic pancreatitis compared with patients with type 2 diabetes and healthy individuals. Diabetes Care (2017) 40:1314–22. doi: 10.2337/dc17-0792 28751547

[34] Kozawa J Okita K Iwahashi H Yamagata K Imagawa A Shimomura I. Early postprandial glucagon surge affects postprandial glucose levels in obese and non-obese patients with type 2 diabetes. Endocr J (2013) 60:813–8. doi: 10.1507/endocrj.EJ13-0018 23459463

[35] Morettini M Burattini L Göbl C Pacini G Ahrén B Tura A. Mathematical model of glucagon kinetics for the assessment of insulin-mediated glucagon inhibition during an oral glucose tolerance test. Front Endocrinol (Lausanne) (2021) 12:611147. doi: 10.3389/fendo.2021.611147 33828527 PMC8020816

[36] Robertson RP. Brief overview: glucagon history and physiology. J Endocrinol (2023) 258. doi: 10.1530/JOE-22-0224 37227172

[37] Lund A Bagger JI Albrechtsen NJW Christensen M Grøndahl M Hartmann B. Evidence of extrapancreatic glucagon secretion in man. Diabetes (2016) 65:585–97. doi: 10.2337/db15-1541 26672094

[38] Ahrén B. Glucagon - Early breakthroughs and recent discoveries. Peptides (2015) 67:74–81. doi: 10.1016/j.peptides.2015.03.011 25814364

[39] Færch K Vistisen D Pacini G Torekov SS Johansen NB Witte DR. Insulin resistance is accompanied by increased fasting glucagon and delayed glucagon suppression in individuals with normal and impaired glucose regulation. Diabetes (2016) 65:3473–81. doi: 10.2337/db16-0240 27504013

[40] Kattner N Al-Selwi Y Tiniakos D Klöppel G Shaw JA. 1451-P: peri-islet fibrosis in cystic fibrosis is associated with altered alpha-cell phenotype. Diabetes (2022) 71:1451–P. doi: 10.2337/db22-1451-P

