# Supplementary material for: Characterization of impaired beta and alpha cell function in response to an oral glucose challenge in cystic fibrosis: a cross-sectional study
Source: Front Endocrinol (Lausanne). 2023 Aug 31;14:1249876. doi: 10.3389/fendo.2023.1249876 (PMC10501799; doi:10.3389/fendo.2023.1249876)
Supplement: Supplementary file 1 [file DataSheet_1.docx]

Supplementary material

**Supplementary Figure 1. Time to nadir glucagon (T_Min_) concentration by glucose tolerance group during an oral glucose tolerance test among 87 adults with cystic fibrosis**

*
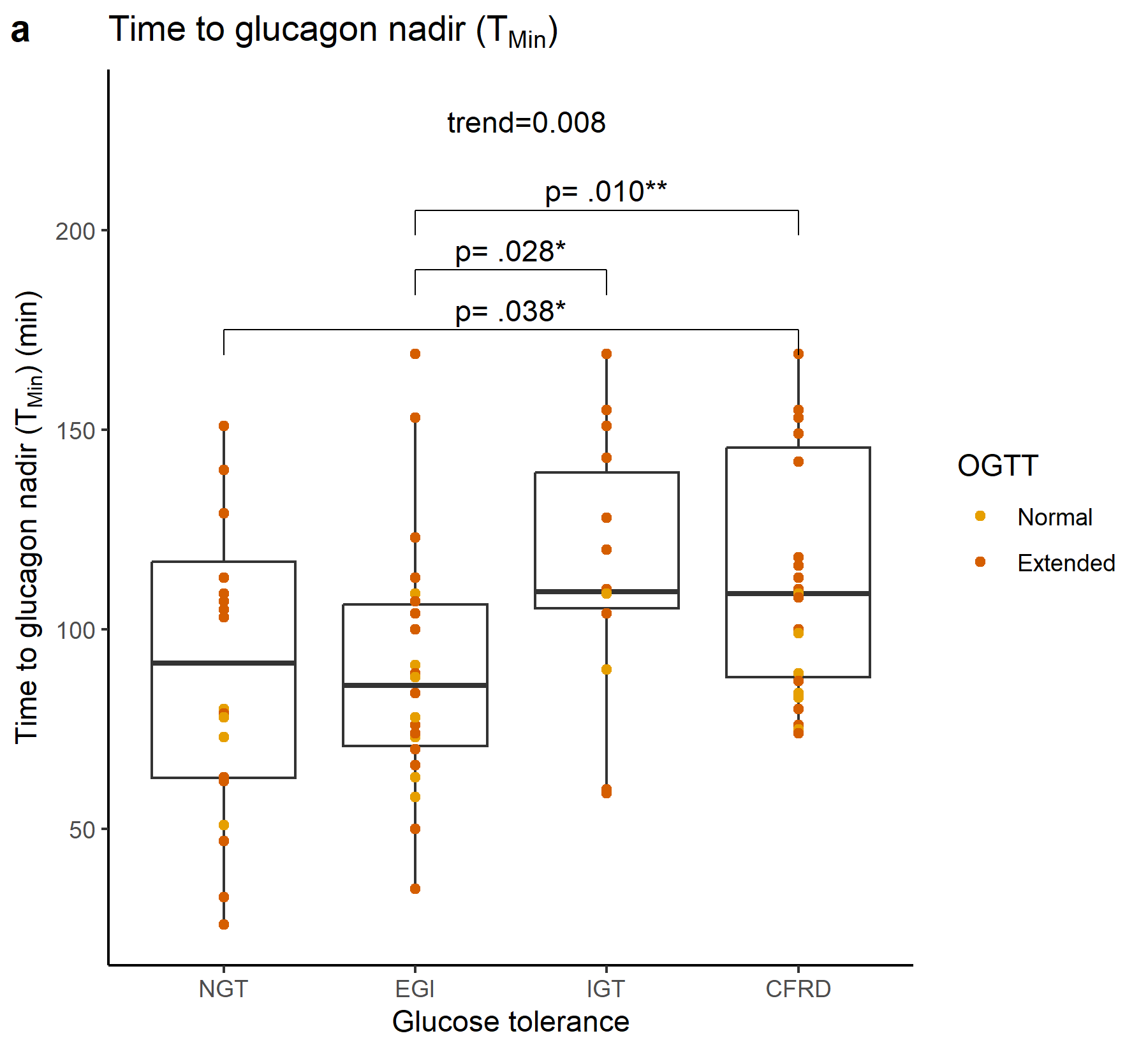
*

*Time to nadir (minimum) glucagon concentration by glucose tolerance group compared using a linear regression model with robust standard error. Yellow scatters were estimated from a standard oral glucose tolerance test with 4 time points (-1, 30, 60, and 120 min) and orange scatters were estimated from an extended oral glucose tolerance test with 11 time points. Glucagon was not assessed in six individuals due to delivery failure of the samples. NGT, normal glucose tolerance; EGI, early glucose intolerance; IGT, impaired glucose tolerance; CFRD, cystic fibrosis-related diabetes; OGTT, oral glucose tolerance test.*

**Supplementary Figure 2. Correlations between parameters derived from a standard (4 time points) oral glucose tolerance test and the parameters derived from all available time points during an extended or standard oral glucose tolerance test**

*
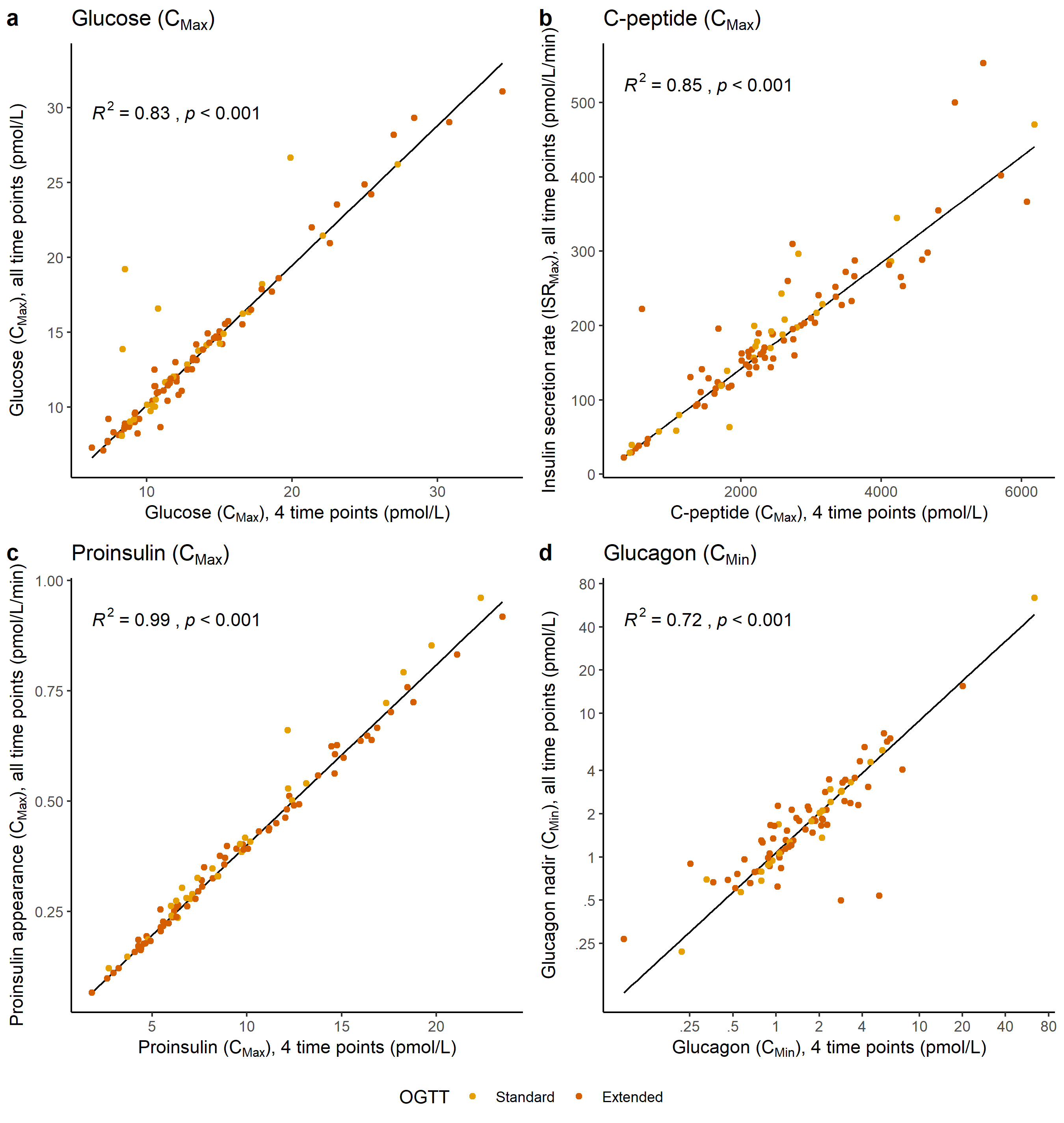
*

*Spearman’s correlations were used to validate the use of simple parameters derived from 4 time points (C_Max_ and C_Min_ after cubic spline interpolation between -1, 30, 60, and 120 min) compared to advanced parameters derived from all available time points (a-b: minimal model adapted to oral glucose tolerance tests by Breda et al., c: deconvolution after cubic spline interpolation, d: minimum measured value). Glucose (a), C-peptide (b), proinsulin (c) and glucagon (d) are depicted with yellow scatters if only 4 time points (standard OGTT) were available and with orange scatters if all 11 time points (extended OGTT) were collected during an oral glucose tolerance test.*

*Abbreviations: ISR_Max_, Maximum insulin secretion rate; C_Max_, maximum concentration; C_Min_, minimum/nadir concentration; OGTT, oral glucose tolerance test.*

**Supplementary Table 1. Pancreas hormones in participants with level 2 hypoglycemia (n=4) reported as percentage difference relative to participants with non-hypoglycemia (n= 33) in adults with cystic fibrosis and either normal glucose tolerance or early glucose intolerance during an extended oral glucose tolerance test**

|  |  | **Adjusted** | | | |
| --- | --- | --- | --- | --- | --- |
|  | **n** | **%** | **95% CI** | | **p-value** |
| **Glucose (mmol/L)** |  |  |  |  |  |
| 60 min | 37 | 23.4 | -3.4 | 57.5 | 0.092 |
| 120 min | 37 | -10.1 | -29.6 | 14.7 | 0.391 |
| 180 min | 37 | -43.3 | -55.6 | -27.6 | **<0.001** |
| **Insulin (pmol/L)** |  |  |  |  |  |
| 60 min | 37 | 31.8 | -37.2 | 176.6 | 0.465 |
| 120 min | 37 | 63.6 | -21.8 | 242.4 | 0.191 |
| 180 min | 37 | -23.4 | -65.1 | 68.0 | 0.505 |
| **C-peptide (pmol/L)** |  |  |  |  |  |
| 60 min | 37 | 16.1 | -27.7 | 86.3 | 0.536 |
| 120 min | 37 | 51.3 | -5.6 | 142.6 | 0.085 |
| 180 min | 37 | 15.8 | -27.8 | 85.7 | 0.543 |
| **Proinsulin (pmol/L)** |  |  |  |  |  |
| 60 min | 37 | -5.4 | -36.5 | 41.0 | 0.786 |
| 120 min | 37 | 15.4 | -22.5 | 71.9 | 0.480 |
| 180 min | 37 | 0.4 | -32.6 | 49.6 | 0.983 |
| **Total proinsulin/insulin ratio (%)** |  |  |  |  |  |
| 0-60 min | 37 | -9.7 | -38.6 | 33.0 | 0.606 |
| 60-120 min | 37 | -25.9 | -49.7 | 9.1 | 0.129 |
| 120-180 min | 37 | -13.7 | -41.3 | 27.1 | 0.456 |
| **Glucagon (pmol/L)** |  |  |  |  |  |
| 60 min | 32 | 159.1 | 6.2 | 532.3 | **0.036** |
| 120 min | 32 | 73.0 | -29.1 | 322.3 | 0.228 |
| 180 min | 32 | 325.3 | 57.7 | 1047.1 | **0.004** |

*Percentage difference in pancreas hormones in participants with level 2 hypoglycemia (glucose_180 min_<3.0 mmol/L) relative to participants with non-hypoglycemia. Data is based on back transformed beta coefficients (% and 95% confidence intervals) estimated with linear mixed models with time as factor and logarithmic transformed glucose concentrations/pancreas hormones. The model included robust standard errors and were adjusted for age, sex, body mass index and exocrine pancreas function. Total proinsulin/insulin ratio was calculated using the area under the curve of proinsulin appearance and insulin secretion rate. Glucagon was not assessed in five individuals due to delivery failure of the samples.*
